# Mental Disorders in patients hospitalized due to Neurologic Disorders: a nationwide study

**DOI:** 10.1192/j.eurpsy.2024.186

**Published:** 2024-08-27

**Authors:** M. Gonçalves-Pinho, B. Martins, A. Costa, J. Pedro Ribeiro, A. Freitas, E. Azevedo, L. Fernandes

**Affiliations:** ^1^CINTESIS@RISE, Department of Clinical Neurosciences and Mental Health, Faculty of Medicine, University of Porto, FMUP, Porto; ^2^CHTS, Penafiel; ^3^Department of Clinical Neurosciences and Mental Health, Faculty of Medicine, University of Porto; ^4^CINTESIS@RISE, MEDCIDS, Faculty of Medicine, University of Porto, FMUP; ^5^Psychiatry Service - Centro Hospitalar Universitário de São João, CHUSJ, Porto, Portugal

## Abstract

**Introduction:**

The presence of psychiatric comorbidity significantly impacts the quality of life for patients and often goes unnoticed within the realm of neurology.

**Objectives:**

This study’s objective was to elucidate and characterize psychiatric comorbidity among patients hospitalized for neurological disorders in mainland Portugal.

**Methods:**

This retrospective observational study analyzed hospitalizations categorized with a primary diagnosis of neurological disorders, defined by Clinical Classification Software (CSS) for ICD-9-CM codes 76, 77, 79-85, 95, and 109, occurring in adult patients (≥18 years) between 2008 and 2015. Psychiatric comorbidity was determined by the presence of secondary diagnoses falling under CCS categories 650-670.

**Results:**

A total of 294,806 hospitalization episodes were documented with a primary diagnosis of neurological disorders in adult patients between 2008 and 2015 in Portuguese public hospitals. Approximately 26.9% (n=79,442) of these episodes were associated with documented psychiatric comorbidity (22.1% for female hospitalizations and 32.2% for male hospitalizations). Patients with recorded psychiatric comorbidity were younger (66.2±16.2 vs. 68.6±17.2 for those without psychiatric comorbidity, p<0.001), exhibited a lower overall in-hospital mortality rate, and experienced significantly longer mean hospital stays. Among these comorbidities, ‘Delirium, dementia, amnestic, and other cognitive disorders’ were documented in 7.4% (n=21,965) of hospitalizations, followed by alcohol-related disorders in 6.5% (n=19,302) and mood disorders in 6.1% (n=18,079). Epilepsy/seizures had the highest recorded psychiatric comorbidity rate among neurological disorders (39.9%).

**Conclusions:**

Psychiatric comorbidity is present in more than a quarter of hospitalizations with a primary diagnosis of neurological disorders. The prevalence of psychiatric comorbidity varies across different neurological disorders and is associated with distinct demographic and clinical characteristics.

**Disclosure of Interest:**

None Declared

